# Covalent triazine framework modified with coordinatively-unsaturated Co or Ni atoms for CO_2_ electrochemical reduction†
†Electronic supplementary information (ESI) available. See DOI: 10.1039/c8sc00604k


**DOI:** 10.1039/c8sc00604k

**Published:** 2018-03-19

**Authors:** Panpan Su, Kazuyuki Iwase, Takashi Harada, Kazuhide Kamiya, Shuji Nakanishi

**Affiliations:** a Research Center for Solar Energy Chemistry , Osaka University , 1-3 Machikaneyama, Toyonaka , Osaka 560-8531 , Japan . Email: kamiya@chem.es.osaka-u.ac.jp ; Email: nakanishi@chem.es.osaka-u.ac.jp; b Department of Applied Chemistry , The University of Tokyo , 7-3-1 Hongo, Bunkyo-ku , Tokyo 113-8656 , Japan; c Graduate School of Engineering Science , Osaka University , 1-3 Machikaneyama, Toyonaka , Osaka 560-8531 , Japan; d Japan Science and Technology Agency (JST) , PRESTO , 4-1-8 Honcho , Kawaguchi , Saitama 332-0012 , Japan

## Abstract

The electrochemical reduction of carbon dioxide (CO_2_) has attracted considerable attention as a means of maintaining the carbon cycle. This process still suffers from poor performance, including low faradaic efficiencies and high overpotential. Herein, we attempted to use coordination number as a control parameter to improve the electrocatalytic performance of metal species that have previously been thought to have no CO_2_ reduction activity. Covalent triazine frameworks (CTF) modified with coordinatively-unsaturated 3d metal atoms (Co, Ni or Cu) were developed for efficient electroreduction of CO_2_. Co-CTF and Ni-CTF materials effectively reduced CO_2_ to CO from –0.5 V *versus* RHE. The faradaic efficiency of the Ni-CTF during CO formation reached 90% at –0.8 V *versus* RHE. The performance of Ni-CTF is much higher than that of the corresponding metal-porphyrin (using tetraphenylporphyrin; TPP). First principles calculations demonstrated that the intermediate species (adsorbed COOH) was stabilized on the metal atoms in the CTF due to the low-coordination structure of this support. Thus, the free energy barriers for the formation of adsorbed COOH on the metal atoms in the CTF supports were lower than those on the TPP supports.

## Abstract 2

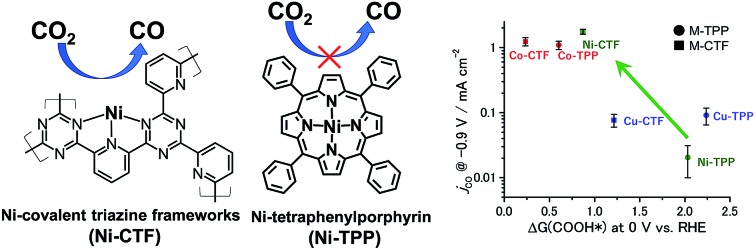
Nickel-modified covalent triazine frameworks effectively reduced CO_2_ to CO because adsorbed COOH was stabilized on the coordinatively-unsaturated Ni atoms in CTF.

## Introduction

The electrochemical carbon dioxide reduction reaction (CO_2_RR) in aqueous media is a promising approach to closing the carbon cycle, and as such has attracted significant attention.^1^ The reduction of CO_2_ to CO summarized in the following equations is an important step in the CO_2_RR and represents the first two-electron reaction.^2^

1 CO_2_(g) + * + H^+^(aq) + e^–^ ↔ COOH*

2 COOH* + H^+^(aq) + e^–^ ↔ CO* + H_2_O

3 CO* ↔ CO(g) + *

here, the asterisk represents a free adsorption site. Previous studies have shown that either reaction (1) or (3) can become the rate-determining step, depending on the relative magnitudes of the COOH* and CO* adsorption energies.2e–g Importantly, there is a linear relationship (scaling relation) between the COOH and CO adsorption energies, such that these values do not change independently of one another.2e,f For this reason, the adsorption energy of COOH* (or CO*) can serve as an indicator of the CO generation activity, and a so-called volcano-type relationship has been established between the adsorption energy and the catalytic activity.2e,f Therefore, developing a highly efficient CO generating electrocatalyst requires precise tuning of the adsorption energies of critical intermediate species.

There are two important factors determining the adsorption energies of intermediates on electrocatalysts. The first is the metal species in the catalyst *i.e.* transition metals at active sites having a greater number of d-electrons tend to lower the adsorption energy.^3^ For this reason, the effects of metal species on the CO_2_RR have been systematically investigated using tetraphenylporphyrin (TPP),2c,^4^ which can serve as a platform to support various metals *via* coordination bonds. It is known that cobalt modified-TPP (Co-TPP) has an appropriate bond strength with COOH* and thus shows efficient CO_2_RR activity, whereas the catalytic activities of nickel (Ni) or copper (Cu) modified-TPP are low because the COOH* binding energy is insufficient.4c–e The second critical factor is the coordination structure (or the coordination number) of the metal.^5^ First principles calculations have demonstrated that a decrease in coordination number of bulk Pt or Cu leads to an increase in the CO* binding strength,5a,b thus affecting the CO_2_RR activity. Therefore, it may be possible to use the coordination number as a control parameter to improve the performance of metal species that have previously been thought to have no CO_2_RR activity.

Covalent triazine frameworks (CTF), consisting of microporous conjugated polymers with 1,3,5-triazine linker units,^6^ are promising platforms for metal species with coordination unsaturation. Our group has previously reported that the adsorption energies of dioxygen and nitrogen oxides on Cu-CTF are larger than those on Cu-TPP or bulk Cu, respectively.^7^ This increase in the adsorption energy was attributed to the coordinatively unsaturated nature of the Cu atoms in the CTF. In the present work, we synthesized CTFs modified with coordinatively-unsaturated 3d metal atoms as electrocatalysts for CO_2_RR with the aim of eliciting the potential performance of metals that have, to date, been regarded as inactive.

## Results and discussion

The CTF was synthesized *via* the polymerization of 2,6-dicyanopyridine in the presence of conductive carbon particles (CPs; KetchenBlack EC600JD, please see the synthesis part) in the same manner as described in our previous reports.^7^,^8^ The obtained CTF was subsequently impregnated with aqueous solutions of various divalent metal chlorides to obtain M(ii)-CTF (M = Co, Ni or Cu). The full details of the synthesis are provided in the Experimental section. The elemental analyses were conducted by semi-quantitative X-ray photoelectron spectroscopy (XPS) and inductively coupled plasma atomic emission spectroscopy (ICP-AES) (Tables S1 and S2†). The CTF layer was uniformly polymerized on CPs, which has been proven by N 1s XPS spectra and transmission electron microscopy (TEM) energy dispersive X-ray (EDX) mapping in our previous reports.8*b* In addition, the SEM pictures for CTF (Fig. S1†) show that the particle size of 20–80 nm, which corresponded to the size of the CPs, also revealed that CTF was well mixed with CPs.^7^,^8^ The powder XRD patterns for CTF (Fig. S2†) indicated that our M-CTFs have amorphous structure, which is consistent with the reported CTF originated from 2,6-dicyanopyridine.6a,d The high-resolution TEM images (Fig. S3†) suggested that no metal or metal oxide nanoparticles was formed on M-CTF, which are consistent with the previous results.7a,8b The nitrogen adsorption–desorption isotherms indicates that CTF and Ni-CTF have a hierarchical pore system composed of micro- and mesopores (see Fig. S4† for the detail).

Electronic and structural characterizations of the metal sites in M-CTF specimens were conducted using extended X-ray absorption fine structure (EXAFS) analyses. Previously, we demonstrated that the Cu atoms in Cu-CTF are in the form of Cu(ii) and have a lower first coordination number of 3.4 compared to the value of 4 in Cu-TPP.7*b* The present study focused on the characterization of Ni-CTF (as presented herein) and Co-CTF (as presented in ESI, see Fig. S6† and the associated caption). Fig. S5† shows the Ni–K XANES spectra for Ni-CTF and the reference samples. The XANES absorption edge corresponding to 1s → 4p transitions^9^ for Ni-CTF located at 8333 eV, which is consistent with that for NiO and Ni(ii)-TPP. These results indicated that the Ni(ii) valence state was dominant in Ni-CTF. Similarly, Fig. S6a† indicated that the Co(ii) valence state was dominant in Co-CTF.

**Fig. 1:**
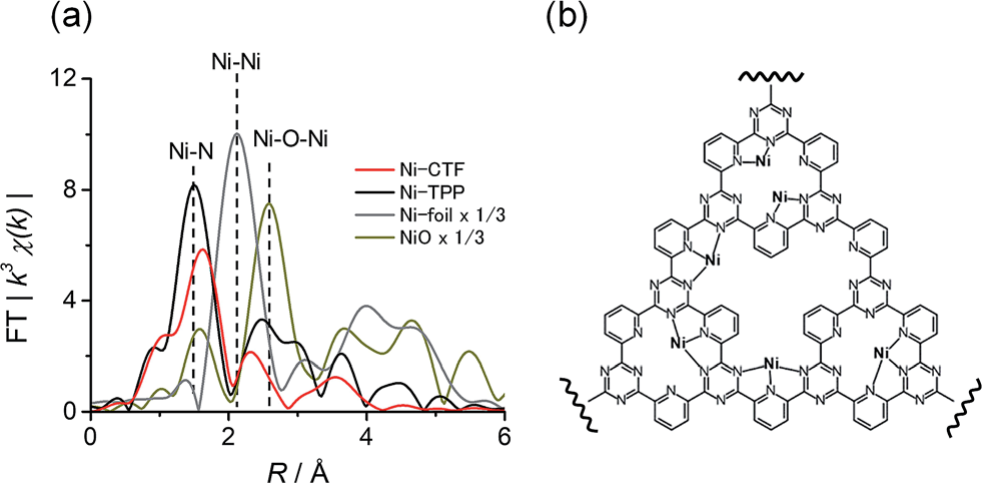
(a) The *k*^3^-weighted Fourier transform EXAFS spectra at the Ni K-edge of Ni-CTF (red), Ni-TPP (black), Ni foil (gray) and NiO (dark yellow), and (b) an illustration showing the Ni sites in Ni-CTF (weakly adsorbed molecules in electrolyte, such as water and ethanol used for the preparation of electrode, are not shown for clarity).

We also acquired EXAFS data to assess the molecular structure of the Ni-CTF (Fig. 1a). Ni-CTF shows one strong peak at 1.7 Å, whereas no strong peak corresponds to Ni–Ni bond and Ni–O–Ni bonds were not observed.^10^ We previously demonstrated that no metal species were found on CPs that were treated with MCl_2_ solutions,7*a* indicating that metal species were anchored on CTFs. In addition, the TEM results showed that no NiO nanoparticle was formed. Thus, the first coordination sphere (the peak at 1.7 Å) was composed of N atoms of CTF. The curve fitting for EXAFS spectra were performed for Ni-CTF (detail see Table S3† and its captions). The coordination number of N atoms in the first coordination sphere of Ni-CTF is 3.4. These data indicate that the Ni atoms in the Ni-CTF were individually isolated and had an unsaturated first coordination sphere in the pores of the CTF (Fig. 1b), identical result is also obtained for Co-CTF (Fig. S6†).

**Fig. 2:**
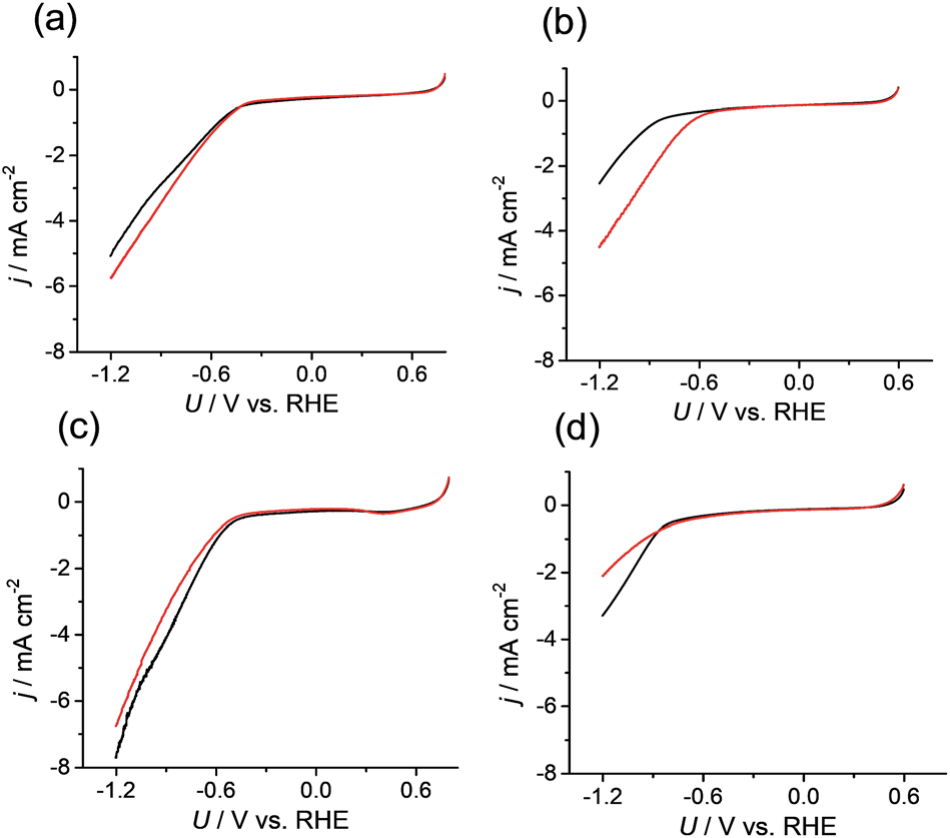
Current density (*j*) *versus* potential (*U*) curves obtained from (a) Co-CTF, (b) Ni-CTF, (c) Cu-CTF, and (d) CTF in a phosphate buffer (saturated with Ar, black line) and a KHCO_3_ electrolyte (saturated with CO_2_, red line).

Because the data confirmed that the metal centers in the M-CTF had lower coordination numbers than those in the corresponding M-TPP, we next evaluated and compared the CO_2_RR activities of these samples. Changes in current density (*j*) at different potentials (*U*) were examined using M-CTF electrodes in Ar-saturated phosphate buffer solutions (PBS, pH 6.8) and CO_2_-saturated KHCO_3_ solutions (pH 6.8). As shown in Fig. 2b, the cathodic current produced by the Ni-CTF was significantly increased when CO_2_ was present in the electrolyte. The onset potential for the reduction reaction in CO_2_-saturated solutions was –0.48 V *versus* RHE, whereas a current attributed solely to the hydrogen evolution reaction (HER) was observed from –0.72 V *vs.* RHE in the absence of CO_2_. In contrast, little or no increase in the reduction current in the presence of CO_2_ was observed when using the Co-CTF, Cu-CTF or bare CTF (Fig. 2b, c and d, respectively). The reduction peak of metal centers was not observed for M-CTF in the absence of CO_2_. This is likely because these peaks were overlapped with the HER current.

**Fig. 3:**
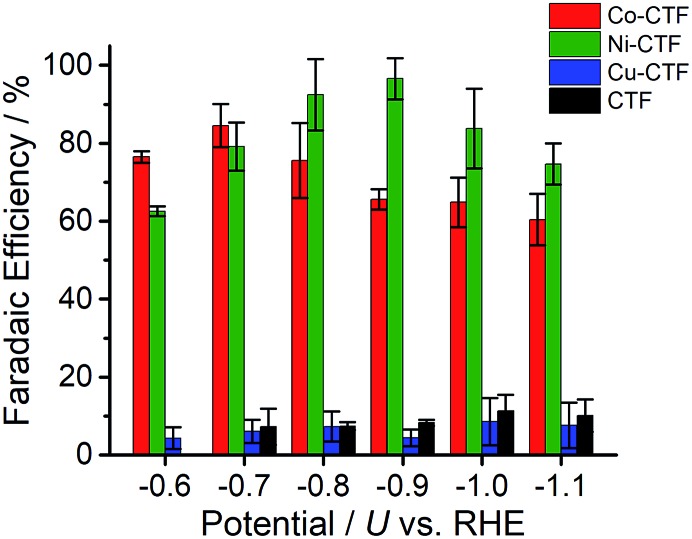
Effect of potential on the faradic efficiency values during the CO_2_RR to generate CO using Co-CTF (red), Ni-CTF (green), Cu-CTF (blue) and CTF (black) in a KHCO_3_ electrolyte (saturated with CO_2_) at pH 6.8. The error bar represents the standard deviation from three experimental trial.

**Fig. 4:**
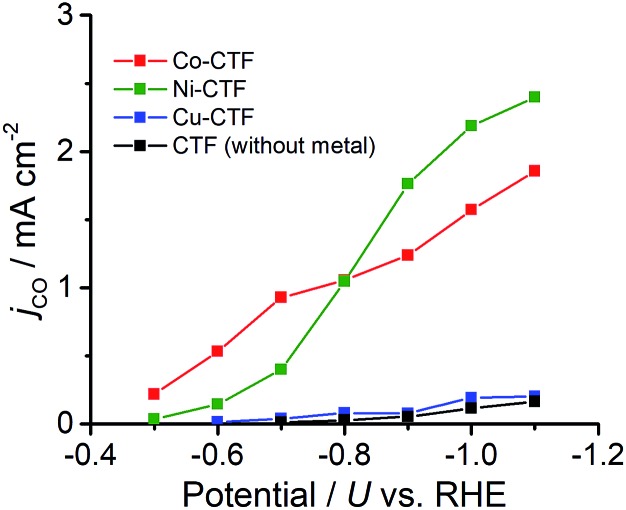
Partial current density values during the CO generation reaction using Co-CTF (red), Ni-CTF (green), Cu-CTF (blue) and CTF (black).

CVs alone cannot provide conclusive evidence for CO_2_ reduction because the different electrolytes (PBS and KHCO_3_ solutions) were used for the comparison. Thus, subsequently, we quantitatively analysed the CO_2_ reduction products in the gas phase using gas chromatography-mass spectrometry (GC-MS). A representative GC-MS chart is shown in Fig. S7.† The effects of potential on the faradaic efficiency (FE) of the CO generation reaction using M-CTFs in neutral solutions are summarized in Fig. 3. At the appropriate potential, the main product of the electrolysis in the presence of CO_2_ when employing either Ni-CTF or Co-CTF was CO, whereas almost no CO evolution was observed with the Cu-CTF or bare CTF throughout the entire experimental potential region. In particular, the Ni-CTF exhibited CO formation with a FE exceeding 90% over the range of –0.8 to –0.9 V. It should be noted that the extent of CO evolution by the bare CTF was almost negligible, indicating that the Ni/Co sites in the CTF served as the catalytic centers. In contrast, the FE of Cu-CTF was similar with that of bare CTF, meaning that the FE of Cu-CTF was affected by the CO_2_RR activity of CTF frameworks to some extent. GC-MS and nuclear magnetic resonance (NMR, Fig. S8†) data showed that H_2_ was the other major product in all cases, along with trace amounts of methane (<0.5%). Note that only H_2_ was observed in the absence of CO_2_, indicating that the CO was generated not from the degradation of CTF but from CO_2_RR (Fig. S7†). The partial current density values (Fig. 4) during the CO generation using Ni-CTF and Co-CTF (3–4 A g^–1^) were equivalent to those reported for a carbon-supported Au catalyst and metal-modified nitrogen-doped carbon catalysts.^11^ The Tafel plots of M-CTF are shown in Fig. S9.† The slope of Tafel plots for Ni-CTF and Co-CTF is 154 mV dec^–1^ and 162 mV dec^–1^, respectively. In contrast, the Tafel slopes for Cu-CTF (305 mV dec^–1^) and CTF (272 mV dec^–1^) were much larger than that of Ni-CTF and Co-CTF, implying the inefficiency of Cu-CTF and CTF for CO_2_ electrochemical reduction. The electrocatalytic stability of Ni-CTF was evaluated at –0.65 V (*vs.* RHE) for 3 h in CO_2_ saturated KHCO_3_ electrolyte. As Fig. S10† shows, the FE for CO displayed almost negligible change, while the reduction current exhibited mildly decreased. As the stability for our catalyst is not enough for the real application, further experiments to improve the stability by choosing the appropriate framework are ongoing in our laboratory.

For comparison purposes, the CO_2_ reduction activities of M-TPP were also tested. In the present work, the M-TPP samples were adsorbed on carbon nanoparticles prior to electro-chemical measurements. An increase in the cathodic current from –0.5 V was observed when using Co-TPP in the presence of CO_2_, whereas the Ni- and Cu-TPP showed no such increase (Fig. S11†). The FE and partial current density values of the CO generation using M-TPP are shown in Fig. S12a and b,† respectively. The partial current density values in association with Co, Ni and Cu-TPP were 1.3, 0.04 and 0.04 mA cm^–2^ at –0.9 V *vs.* RHE, respectively. The major product resulting from the use of Ni- and Cu-TPP was H_2_ at all potentials. Therefore, only Co-TPP exhibited effective CO_2_RR activity among these three TPPs, a result that is consistent with previous studies.4b–e

**Fig. 5:**
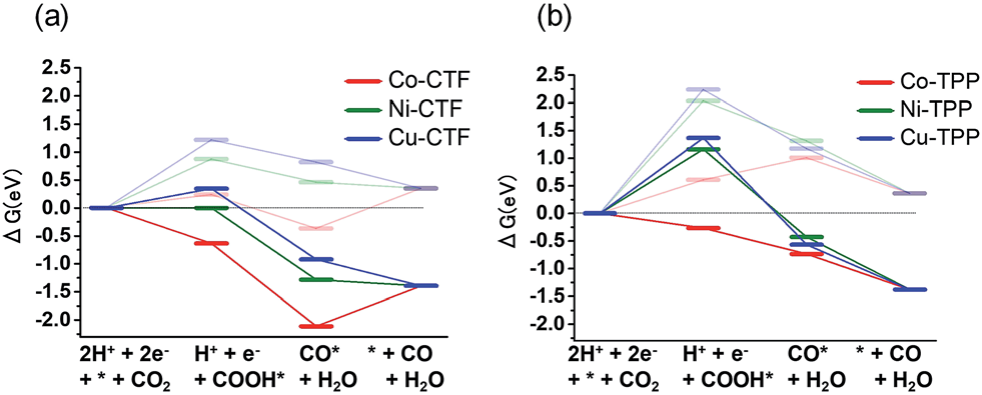
Free energy diagrams for CO_2_ reduction to CO on Co (red), Ni (green) and Cu (blue)-modified (a) CTF, and (b) TPP at 0 V (pale lines) and –0.87 V (dark lines).

The origin of the differences in the activities during CO_2_ reduction to CO of the M-CTF and M-TPP were subsequently examined, employing DFT calculations. After structural optimization of M-CTF, the 3-coordination metal site was the most stable, as represented in Fig. S13† (see the Experimental section for the detail). Thus, the DFT results also revealed that the low coordination metal sites were stabilized by the CTF frameworks, which is basically consistent with the EXFAS results. Fig. 5 and Table S4† shows the free energy diagrams for the electrochemical reduction of CO_2_ to CO on M-CTF and M-TPP at 0 V and –0.87 V *vs.* RHE, based on the computational hydrogen electrode model.2g,11b,^12^ Notably, an increase in the number of d-electrons of the transition metal representing the active sites increased the Gibbs free energy change (Δ*G*) value associated with the formation of COOH* and CO* in the case of both the CTFs and TPPs (although the energy change was in the order of Co < Ni < Cu). These data are consistent with results reported for work with M-porphyrins in association with the CO_2_RR and also the HER and oxygen reduction reaction.^3^,4c,e The rate determining step when using these materials is evidently the first reduction reaction: the formation of COOH* (reaction (1)). The free energy barrier of this step depends on the applied potential,2g,11b,12a and so we calculated the CO_2_ limiting potential (*U*_L_ (CO_2_)) for each catalyst, defined as the potential at which all elementary steps become exergonic, as summarized in Table S5.† The *U*_L_ (CO_2_) obtained with the Co-TPP was approximately –0.6 V *vs.* RHE, whereas the values for the Cu-TPP and Ni-TPP were much more negative (<–2.0 V). Therefore, a low energy barrier to the formation of COOH* apparently resulted in the high CO_2_ reduction activity exhibited by the Co-TPP. In addition, the *U*_L_ (CO_2_) values of the CTFs were smaller than those of the corresponding TPP. In particular, the overall reaction pathway on the Ni-CTF becomes exergonic at –0.87 V *vs.* RHE (Fig. 5a), which essentially coincides with the potential range at which CO formation was observed on the same material. In contrast, the *U*_L_ (CO_2_) of the Cu-CTF (–1.2 V) was 0.33 V more negative, and so the formation of COOH* is still endothermic at –0.87 V on the latter. We expect that Cu-based catalysts with a further low-coordination number (less than 3) exhibit efficient CO_2_RR activity.

**Fig. 6:**
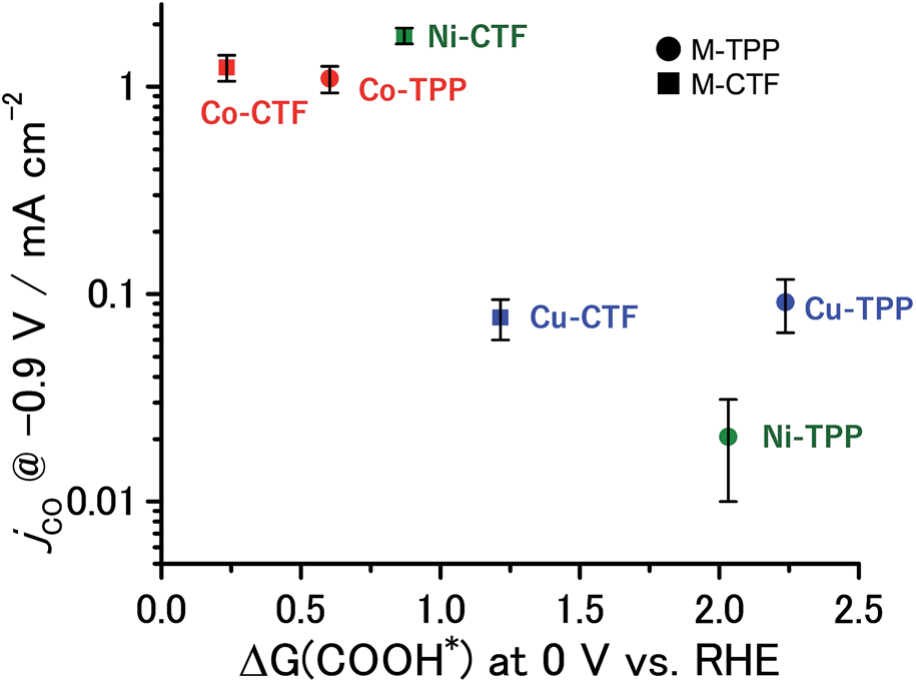
Relationship between Δ*G* (COOH*) at 0 V and *j*_co_ at –0.9 V. The error bar represents the standard deviation from three experimental trial.

The partial current densities during CO generation are plotted against Δ*G* (COOH*) in Fig. 6, clearly indicating that the low Δ*G* (COOH*) is important for high CO_2_RR activity, and also showing that the Ni active sites appear quite high on the plot of CO_2_RR activity upon changing the ligand from TPP to CTF. That is, by transitioning the ligand from TPP to CTF, a lower Δ*G* (COOH*) was obtained such that Ni became the optimal metal species. In the case of the Co-CTF, the release of CO (reaction (3)), which is independent of the applied potential, was an endothermic reaction. As mentioned in the Introduction, the rate-determining step for CO_2_RR, which depends on the adsorption energy of CO and COOH, is the reaction (1) or the reaction (3). However, the existence of the scaling relationship of them does not allow us to independently control these two adsorption energies.2e,f,^13^ Therefore, on the basis of the DFT calculations, there should be the volcano-type relation accompanied with the single peak which locates between Co-CTF and Co-TPP in Fig. 6. The rate-determining step can be evaluated by the position in the volcano plot against the peak. Co-CTF has lower Δ*G* (COOH*) than the optimal value (the peak of the volcano plot), resulting that the rate-determining step was the reaction (3) (CO desorption). Thus, in contrast to Cu-based catalysts, Co based catalysts with a further low-coordination number, which bind CO more strongly, should show poor CO_2_RR activity. In contrast, the rate-determining step of the other catalysts in the higher Δ*G* (COOH*) side was the reaction (1) (CO_2_ activation). To complete the wide range of volcano-type curve, the investigation of CO_2_RR activity of catalysts with different metal species and coordination numbers is under progress in our laboratory.

At this point, we can explain the origin of the lower Δ*G* (COOH) values exhibited by the M-CTF compared to those for the corresponding M-TPP. The EXAFS results demonstrate that the metal atoms in the CTF have unsaturated coordination structures (that is, low coordination numbers, as shown in Fig. 1b), compared with the metals in the porphyrin-like ligands. Calle-Vallejo and co-authors have used first principles calculations to show that metal sites with lower coordination values have higher adsorption energies because they possess numerous accessible d-orbitals as well as low steric hindrance.5a,c,d Therefore, metal atoms in CTF with low coordination numbers can more strongly bind small molecules than those in N_4_ macrocycles, resulting in a low free energy barrier to the first reaction step.

## Conclusions

In the present work, we developed a novel route to precisely tune the adsorption energies of critical intermediate species for efficient CO_2_RR through the modulation of the coordination number of active centers. The Ni and Co-CTF effectively reduced CO_2_ to CO, whereas, among the M-TPP, only the Co-TPP showed any activity. DFT calculations demonstrated that the free energy barrier of the first reduction step, the formation of adsorbed COOH, was decreased by changing the ligands from TPP to CTF. The lower coordination metal sites were stabilized on the CTF due to their rigid framework, allowing COOH* species to be absorbed at these low coordination sites. Consequently, the Ni-based catalyst obtained using CTF rather than TPP ligands was situated at a higher position on the volcano-like CO_2_RR activity plot. Thus, our work provides a new avenue to design efficient catalysts for CO_2_RR.

## Experimental

### Synthesis of CTF

Each CTF was prepared using essentially the same method described in our previous publications.^7^ Briefly, 2,6-dicyanopyridine (64.5 mg, Koei Kagaku) and Ketjen Black EC600JD (64.5 mg, Lion Corp.) were mixed with ZnCl_2_ (6.82 g, Wako) in a glass vacuum tube and then heated to 400 °C at 3.3 °C min^–1^ and held at that temperature for 40 h. The resulting powder was washed with deionized water, tetrahydrofuran (THF, Wako), HCl (1 M, Wako) and aqueous ammonia (1 M, Wako). The obtained CTF was dispersed in a 10 mM aqueous solution of MCl_2_ (M = Co, Ni or Cu) and stirred at 80 °C for 3 h. Subsequently, the product was collected by centrifugation and thoroughly washed with deionized water to remove any unbound metal salt, followed by drying at 60 °C in a vacuum oven for 1 day.

### Loading of M-TPP onto carbon particles

Here, we take Co-TPP electrode as an example to show the preparation procedure of M-TPP electrode. Firstly, Co-TPP (9.5 mg) and EC600JD (90 mg) were dispersed in 20 mL DMF solvent using an ultrasonic bath, and then, the mixture was dried at 80 °C with a rotary evaporator. The metal concentration of the mixture of M-TPP and EC600JD is the same with that of the corresponding M-CTF (determined by the XPS measurements). These loaded M-TPP are denoted as M-TPP.

### Characterizations

XPS (Axis Ultra, Kratos Analytical Co.) spectra were obtained using monochromatic Al Kα X-rays at *hν* = 1486.6 eV. XAFS data were acquired by the transmission method using the hard X-ray BL01B01 beam line at the SPring-8 facility, Japan. Transmitted X-rays were detected using a double-crystal Si (111) monochromator. Surface inspection was carried out with a scanning electron microscope (SEM; JEOL, JSM-7600F) and high-resolution transmission electron microscope (TEM; Hitachi, H-9000NAR). XANES spectra and EXAFS were analysed using Athena, ARTEMIS and FEFF6L software. A powder X-ray diffraction (XRD) pattern was recorded on a PANalytical X'Pert PRO diffractometer with Cu Kα radiation. Metal content was determined by inductively coupled plasma atomic emission spectroscopy (ICP-AES; Optima 8300, PerkinElmer). The N_2_ adsorption–desorption isotherm was obtained by a Micromeritics 3 Flex at 77 K.

### Electrochemical measurements

Electrochemical measurements were performed using a Hokuto Denko Electrochemical Station (Model HZ-5000) in a two-compartment electrochemical cell (separated by a Nafion membrane) in conjunction with three electrodes. A Ag/AgCl electrode (with saturated KCl as the internal solution) and platinum wire were used as the reference and counter electrodes, respectively. Each working electrode was fabricated by dispersing 3 mg of the desired M-CTF (or CTF) and 28.5 μL Nafion (5 wt%, Aldrich) in 300 μL ethanol using an ultrasonic bath to generate a catalyst ink. A 60 μL quantity of this ink was dropped onto a glassy carbon plate (2 cm^2^), which was then left to dry in air at room temperature, to yield a catalyst layer with a loading of 0.3 mg cm^–2^. The gaseous products that accumulated in the cathodic part of the reaction cell were analyzed by GC-MS (GCMS-QP 2010 Plus, Shimadzu, Japan), calibrated using CO and H_2_ samples diluted with air to various concentrations. Products in liquid phase were analyzed on a Varian 500 MHz NMR spectrometer. A 0.5 mL electrolyte (0.1 M KHCO_3_) after electrolysis was mixed with D_2_O (0.1 mL) and dimethyl sulphoxide (32 μL) as internal standard.

### DFT calculations

Density functional theory (DFT) calculations of the adsorption energies of intermediates on the M-CTF and M-TPP were performed using the OpenMX code.^14^ The generalized gradient approximation of the Perdew–Burke–Ernzerhof model (GGA-PBE) was applied, and a kinetic energy cutoff of 120 Ryd was selected. The detailed structural parameters of the M-CTF can be found in our previous publication.7*b* The structure of CTF frameworks without metals was first relaxed. Then, we doped metal sites and relaxed the metal configuration, while the frameworks were fixed. The optimized Ni-CTF structure is shown in Fig. S13.† One COOH or CO molecules were adsorbed on one metal sites, and their configurations were relaxed to calculate the free energy diagram. To simplify the DFT calculations, we used a slab consisting of a single CTF layer as the model structure of M-CTFs. A slab model was used with a 15 Å vacuum layer between the CTF layers. Zero-point energy and entropic corrections were applied based on reported values4c,12a to convert electronic energies into Gibbs free energies. In this work, the solvation energy was not applied and a –0.24 eV correction was added to CO to compensate for a limitation of the PBE model.4c,^15^ Reaction free energies were calculated using the computational hydrogen electrode (CHE) model, following the same method as described in previous reports.2g,11b,12a Briefly, in this model, the chemical potential of a proton–electron pair in solution is equal to half that of a gas phase H_2_ molecule at 0 V *vs.* RHE. Thus, we utilized the following equation to apply the overpotential when calculating the free energy diagram.

4 *G*[H^+^ + e^–^] = 0.5 *G*[H_2_] – *eU*

here, *U* is the applied overpotential and *e* is the elementary charge.

## Conflicts of interest

There are no conflicts to declare.

## Supplementary Material

Supplementary information
