# Supplementary material for: Covalent triazine framework modified with coordinatively-unsaturated Co or Ni atoms for CO2 electrochemical reduction
Source: Chem Sci. 2018 Mar 19;9(16):3941–7. doi: 10.1039/c8sc00604k (PMC5941196; doi:10.1039/c8sc00604k)
Supplement: Supplementary file 1 [file SC-009-C8SC00604K-s001.pdf]

Electronic Supplementary Material (ESI) for Chemical Science.
This journal is © The Royal Society of Chemistry 2018

Supporting Information

Covalent triazine framework modified with coordinatively-unsaturated Co or Ni atoms for CO_2 electrochemical reduction

Panpan Su,[†a] Kazuyuki Iwase,[†b] Takashi Harada,[a] Kazuhide Kamiya,[*a,c,d] and Shuji Nakanishi[*a,c]

Table S1. XPS elemental analysis results (at. %).

	C	O	N	Metal
CTF	86.75	5.93	6.83	-
Ni-CTF	88.29	3.95	7.06	0.14 (Ni)
Co-CTF	89.47	3.09	6.70	0.17 (Co)
Cu-CTF	88.20	3.60	6.74	0.55 (Cu)

Table S2. Metal concentration on M-CTF derived from ICP-OES (at. %).

	Metal
Ni-CTF	0.17 (Ni)
Co-CTF	0.13 (Co)
Cu-CTF	0.38(Cu)

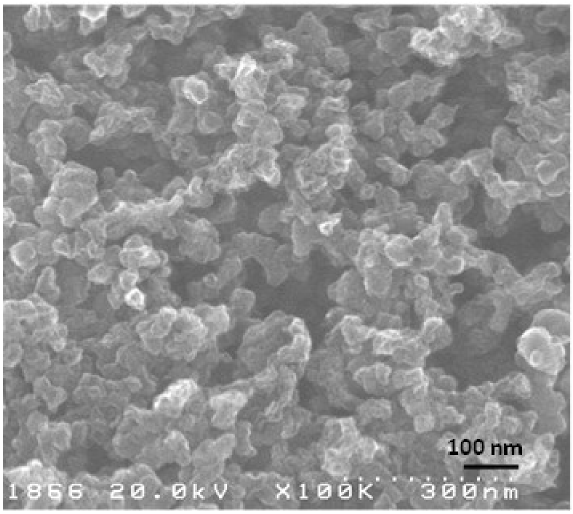

Fig. S1. Representative SEM image of CTF.

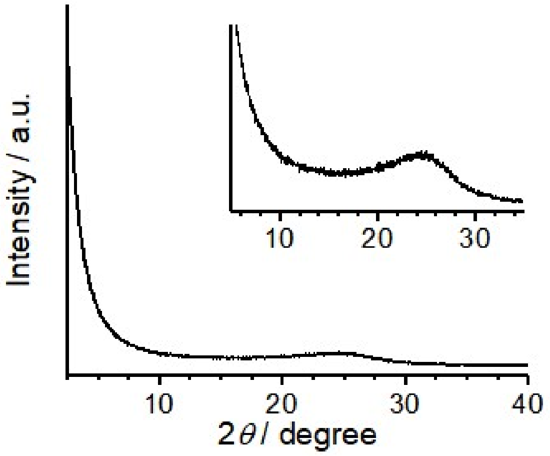

Fig. S2. The XRD pattern of CTF. (inset) magnified one.

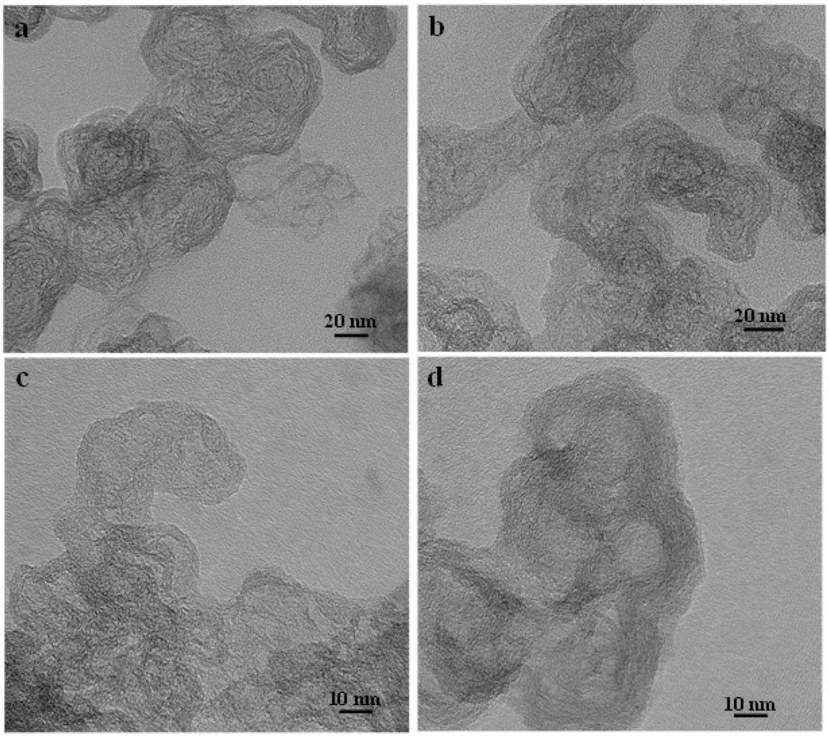

Fig. S3. The TEM images of (a) CTF, (b) Ni-CTF, (c) Co-CTF and (d) Cu-CTF.

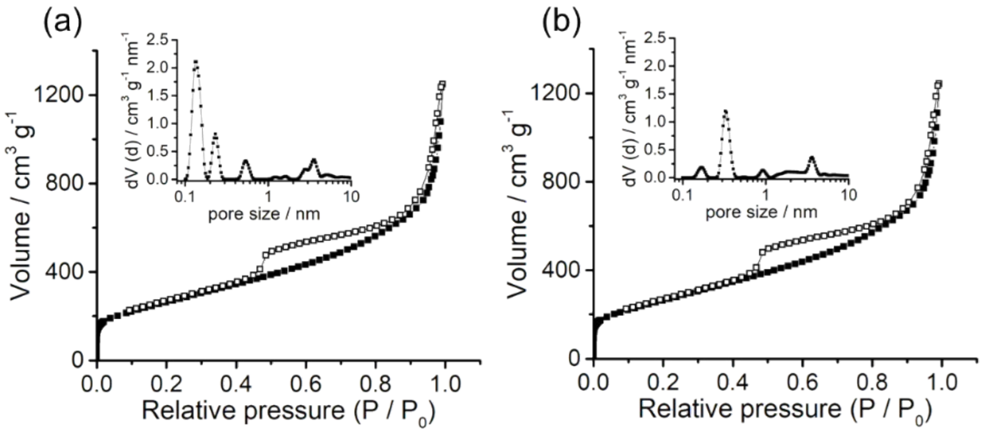

Fig. S4. N_2 adsorption-desorption isotherm for CTF (a) and (b) Ni-CTF. The insets show the pore size distributions calculated by nonlocal density functional theory (NL-DFT) method. BET surface area is 937.5 m^2/g and 933.4 m^2/g for CTF and Ni-CTF, respectively.

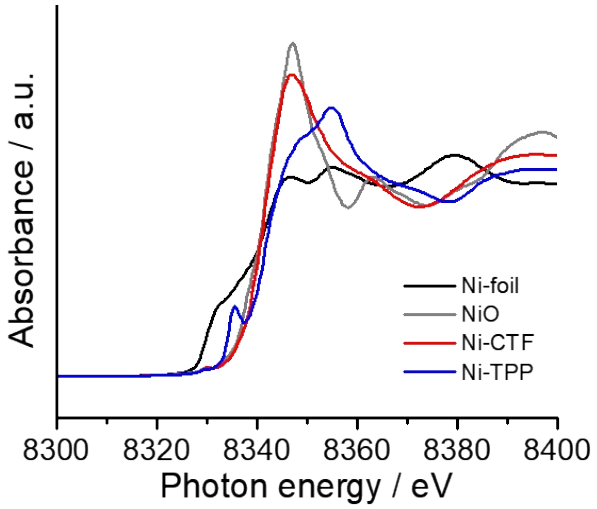

Fig. S5. The Ni-K XANES spectra for Ni-CTF, Ni metal, NiO and Ni(II)-TPP.

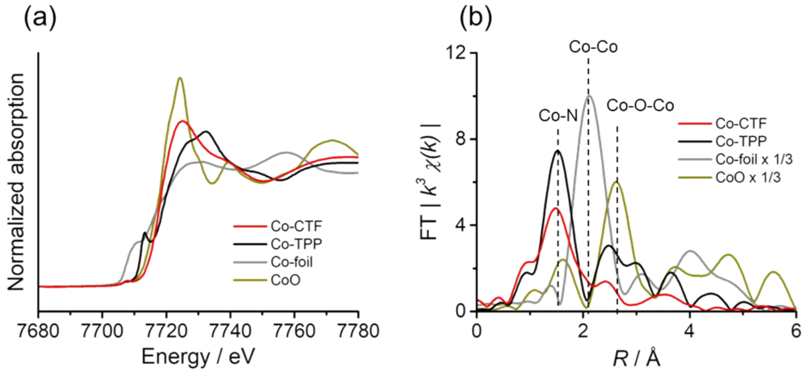

Fig. S6. (a) The Co-K XANES spectra for Co-CTF, Co metal, CoO and Co(II)-TPP, and (b) k^3-weighted Fourier transform EXAFS spectra at the Co K-edge for Co-CTF and Co-TPP.

Figure S6a) shows the Co-K XANES spectra for Co-CTF and the reference samples. The XANES absorption edge corresponding to 1s -> 4p transitions for Co-CTF located at 7710 eV, which is consistent with that for CoO and Co(II)-TPP. These results indicated that the Co(II) valence state was dominant in Co-CTF. The EXAFS results (Figure S6b) indicate that the Co atoms were individually isolated and each had an unsaturated first coordination sphere containing N atoms in the CTF pores, similar to the results obtained with Ni-CTF and Cu-CTF.

Table S3. Results of curve fitting for EXAFS measurement

	Bond type	CN	R	σ^2 ($\times 10^{-3}$, Å)	S_0^2
Co-TPP	Co-N	4	1.95±0.02	3.0±1.6	0.99
Co-CTF	Co-N	3.2	2.00±0.10	7.0±4.2	0.99
Ni-TPP	Ni-N	4	1.94±0.03	3.1±2.1	1.08
Ni-CTF	Ni-N	3.4	2.08±0.05	5.7±1.5	1.08

R : atomic distance (Å), CN : coordination number, σ^2 : Debye–Waller factor, S_0^2 : Amplitude Reduction Factor.

Curve fitting for the EXAFS spectra was conducted by using ARTEMIS software.[S1]

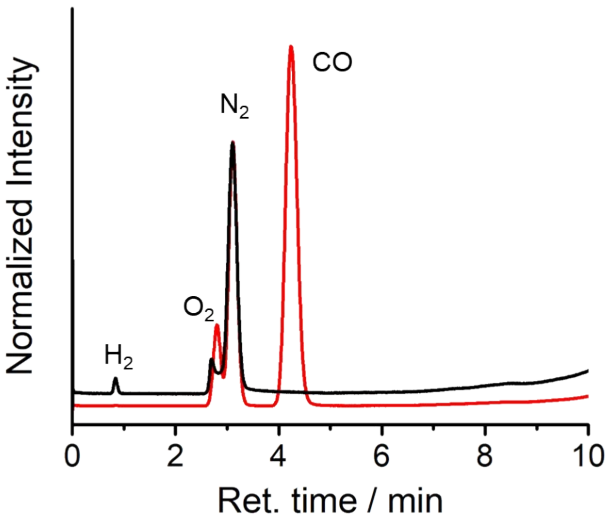

Fig. S7. A representative GC-MS result of Ni-CTF catalyst at -0.9V vs RHE after 3C of electrons flow. Red line; Ni-CTF in $KHCO_3$ solution under CO_2, Black line; Ni-CTF in phosphate buffer solutions under Ar.

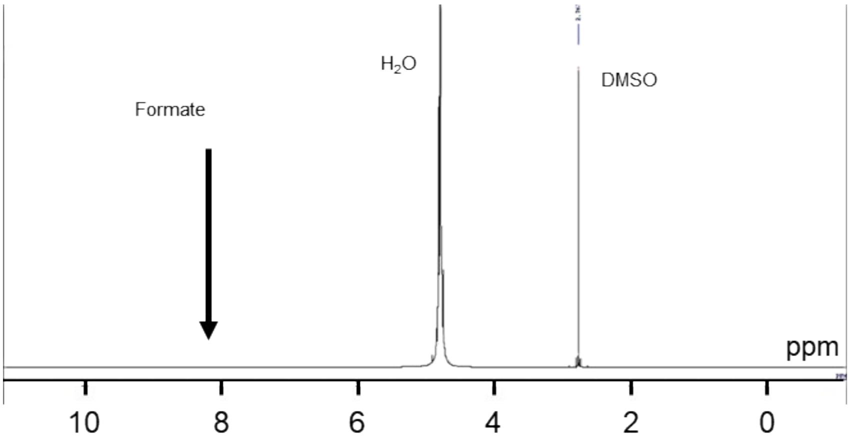

Fig. S8. NMR result of Ni-CTF catalyst at -0.8V vs RHE for 1h. DMSO was added as internal standard.

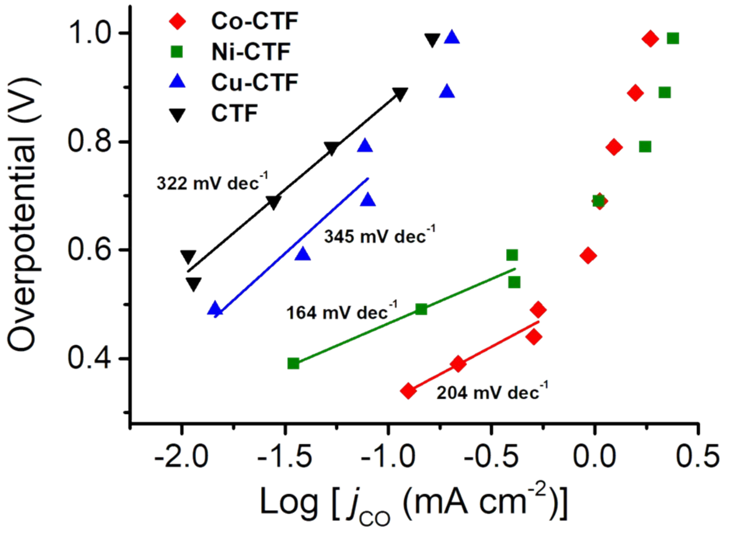

Fig. S9. Tafel plots at different overpotentials as a function with the CO partial current density on M-CTF and CTF catalysts.

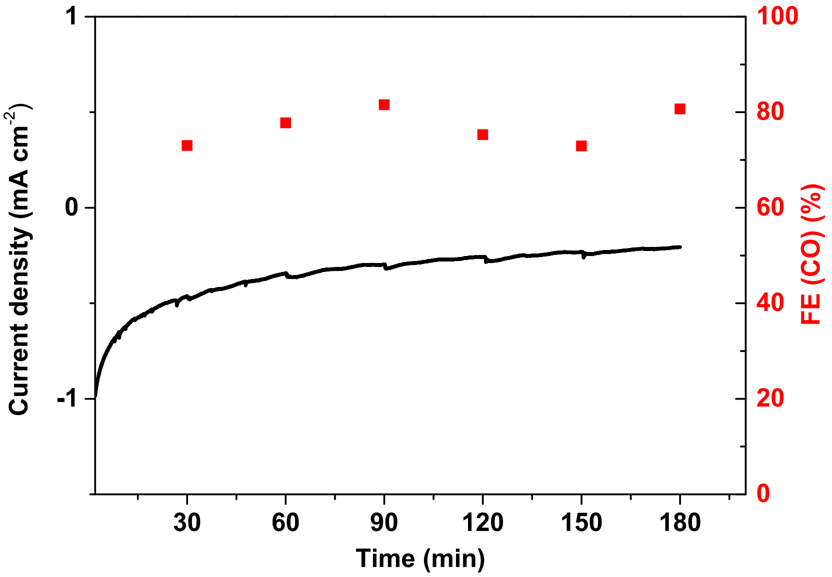

Fig. S10. Chronoamperometric and FE of CO results for the Ni-CTF catalyst in CO_2-saturated $KHCO_3$ (0.1 M) solution at -0.65 V vs RHE for 3h.

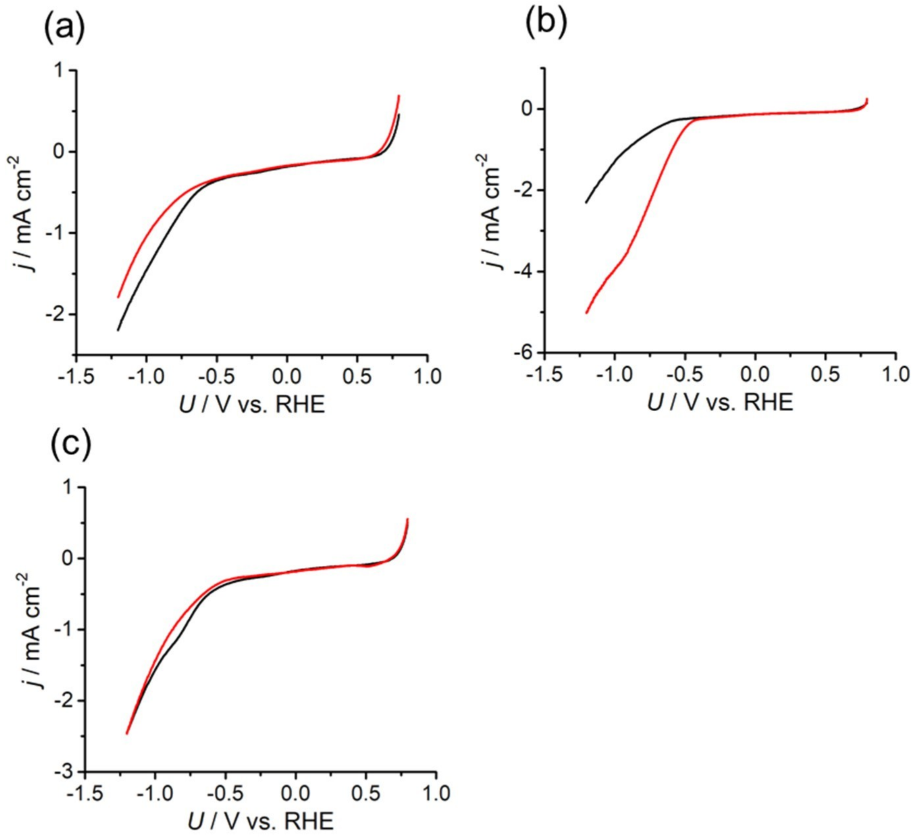

Fig. S11. Current density (*j*) versus potential (*U*) curves obtained from (a) Ni-TPP, (b) Co-TPP, and (c) Cu-TPP in a phosphate buffer (saturated with Ar, black line) or a $KHCO_3$ electrolyte (saturated with CO_2, red line).

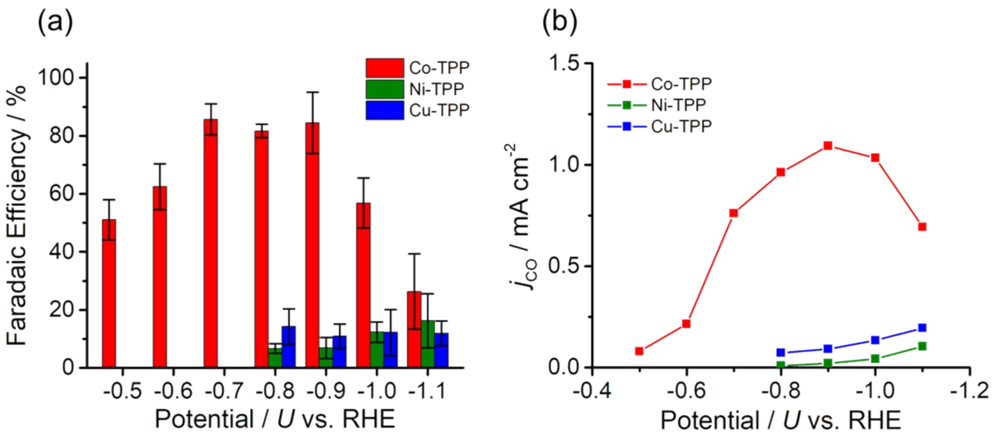

Fig. S12. (a) Faradaic efficiency values and (b) partial current densities during CO generation for Co, Ni and Cu-TPP. The error bar represents the standard deviation from three experimental trial.

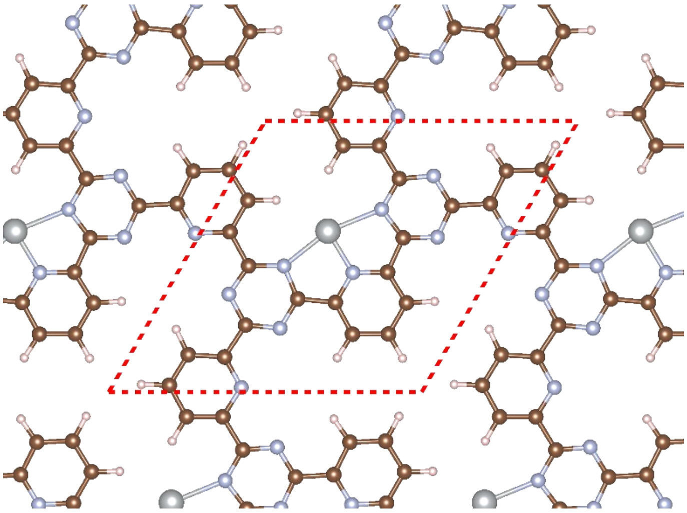

Fig. S13. Optimized structures of metal-CTF (Ni-CTF) for the DFT calculation (brown : C, pink : H, blue : N, gray : Ni). The red dashed line shows the unit cell. Almost the same structure was obtained for Cu- and Co-CTF. Red dashed line represents the unit cell. The detail structural parameters were shown in our previous paper.[S2]

Table S4. Calculated values for free energy diagrams in Figure 5 in the main paper (@ -0.87V).

	CTF / eV		
	Co	Ni	Cu
Step 1	-0.63584	0.000259	0.35071
Step 2	-2.11152	-1.28383	-0.92238
Step 3	-1.39497	-1.39497	-1.39497

TPP / eV		
Co	Ni	Cu
-0.2668	1.162001	1.366827
-0.73393	-0.4277	-0.56717
-1.38032	-1.38032	-1.38032

Table S5. $U_L(CO_2)$ values for M-CTF and M-TPP specimens: the potentials at which all elementary steps become exergonic.

$U_L(CO_2)$ / eV	CTF	TPP
Co	-0.23*	-0.60
Ni	-0.87	-2.03
Cu	-1.22	-2.24

*The reaction (3) on Co-CTF is endothermic.

$U_L(CO_2)$ was calculated as below.[S3] From the reaction (1), we can define ΔG(COOH*).

$$\Delta G(COOH^*)=G[COOH^*]-G[^*]-G[CO_2]-G[H^+ + e^-]$$

$$= G[COOH^*]-G[^*]-G[CO_2] -0.5G [H_2]+eU$$

When ΔG(COOH*) is zero, the U value can be the limiting potential ($U_L(CO_2)$).

Supporting References

S1 B. Ravel, M. Newville *J. Synchrotron Radiat.* **2005**, *12*, 537.

S2 T. Yoshioka, K. Iwase, S. Nakanishi, K. Hashimoto and K. Kamiya, *J. Phys. Chem. C,* **2016**, 120, 15729.

S3 (a) H. Mistry, R. Reske, Z. Zeng, Z.-J. Zhao, J. Greeley, P. Strasser and B. R. Cuenya, *J. Am. Chem. Soc.,* **2014**, 136, 16473. (b) H. A. Hansen, J. B. Varley, A. A. Peterson and J. K. Nørskov *J. Phys. Chem. Lett.* **2013**, 4, 388.
